# The Pass-on Effect of Tetracycline-Induced Honey Bee (*Apis mellifera*) Gut Community Dysbiosis

**DOI:** 10.3389/fmicb.2021.781746

**Published:** 2022-01-18

**Authors:** Shuo Jia, Yuqi Wu, Gongwen Chen, Shuai Wang, Fuliang Hu, Huoqing Zheng

**Affiliations:** College of Animal Sciences, Zhejiang University, Hangzhou, China

**Keywords:** *Apis mellifera*, gut community dysbiosis, tetracycline, pass-on effect, antibiotic residue

## Abstract

Gut microbial community plays an important role in the regulation of insect health. Antibiotic treatment is powerful to fight bacterial infections, while it also causes collateral damage to gut microbiome, which may have long-lasting consequences for host health. However, current studies on honey bees mainly focus on the impact of direct exposure to antibiotics on individual bees, and little is known about the impact of social transmission of antibiotic-induced gut community disorder in honey bee colonies. In order to provide insight into the potential pass-on effect of antibiotic-induced dysbiosis, we colonized newly emerged germ-free workers with either normal or tetracycline-treated gut community and analyzed the gut bacteria composition. We also treated workers with low dosage of tetracycline to evaluate its impact on honey bee gut microbiota. Our results showed that the tetracycline-treated gut community caused disruption of gut community in their receivers, while the direct exposure to the low dosage of tetracycline had no significant effect. In addition, no significant difference was observed on the mortality rate of *A. mellifera* workers with different treatments. These results suggest that though the residue of antibiotic treatment may not have direct effect on honey bee gut community, the gut microbiota dysbiosis caused by high dosage of antibiotic treatment has a cascade effect on the gut community of the nestmates in honeybee colonies.

## Introduction

Honey bee (*Apis* spp.) is one of the most important insect pollinators, contributing an estimated 153 billion euros to the world’s agriculture in 2005 (Gallai et al., 2009). The elevated loss of honey bee colonies around the world (Van der Zee et al., 2015; Brodschneider et al., 2016, 2018) poses a great threat to the food security of the world. Besides pathogens, parasites, nutritional and environmental factors, gut microbiota has been recently proved to be another important factor associated with honey bee health (Clemente et al., 2012; Brodschneider et al., 2018). In contrast to many other animals, honey bees have a relatively simple and conservative gut microbiota community which is dominated by only eight core bacterial phylotypes, including *Snodgrassella alvi*, *Gilliamella apicola*, *Lactobacillus* spp., *Bifidobacterium* spp., *Frischella perrara*, *Bartonella apis*, *Parasaccharibacter apium* and *Commensalibacter spp.* (Kwong et al., 2017a). These gut bacteria contribute to honey bee health in multiple ways, e.g., regulating host hormonal signaling (Zheng et al., 2017), participating in food digestion (Kesnerova et al., 2017; Zheng et al., 2019), priming the immune system against pathogenic infections (Emery et al., 2017; Kwong et al., 2017b) and promoting the endogenous detoxification (Wu et al., 2020b).

Antibiotics are used in beekeeping practice to treat or prevent bacterial infections (Piva et al., 2020), such as American foulbrood disease and European foulbrood disease, which are caused by *Paenibacillus larvae* and *Melissococcus pluton* (Arbia and Babbay, 2011), respectively. However, it has been revealed that the application of tetracycline resulted in major changes in community size structure and led to decreased survivorship of honeybees (Raymann et al., 2017, 2018). Recently, Li et al. (2017) revealed that penicillin-streptomycin treatment weakened bacterial activity in honeybees, which may negatively affect expression levels of genes encoding antimicrobial peptides and increase the honey bee’s vulnerability to *Nosema* infection. These studies provided important insights into how antibiotic treatment affects honey bee gut community and health, while they mainly focused on the direct impacts on the individuals that received the antibiotic treatment. As a social insect, honey bee colony is rooted in a fundamental biological phenomenon known as superorganism resilience, that is the ability of a colony to sustain the loss or impairment of individuals without compromising its overall health (Sponsler and Johnson, 2017). For example, Henry et al. (2015) found that honey bee colonies exposed to neonicotinoid-treated oilseed rape suffered an increased mortality rate in the adult, but prevented any detec e change in overall colony growth or honey storage by producing more new workers. Thus, it is vital to investigate the possible pass-on effect of the impact of antibiotics on honey bee gut community, especially on the development of gut community of their nestmates.

The main social transmission routes of honeybee gut microbiota include fecal-oral transmission, oral-oral transmission and contact with the hive components (comb, honey, and beebread), of which fecal-oral transmission is the most important route for the establishment of a typical gut microbiota community in the receiver (Powell et al., 2014). In this study, we evaluated the possible social transmission of the effects of antibiotic treatment from two aspects. Firstly, the pass-on effects of antibiotic treatment on honeybee gut microbiota were investigated *via* feeding newly emerged workers with antibiotic-treated gut community. Secondly, given that the antibiotic residue after the treatment (Matsuka and Nakamura, 1990; Martel et al., 2006) may also negatively influence the colonization of normal gut microbiota, we applied a field relative level of tetracycline (10 μg/mL) to newly emerged workers and investigated its possible impact on the establishment of honey bee gut microbiota. In addition, we also monitored the mortality of honey bee workers to reveal any potential effect of the treatments on honey bee physiology.

## Materials and Methods

### Rearing of Honey Bee

Honey bees (*Apis mellifera*) were kept in the experimental apiary of the Honey Bee Research Laboratory, College of Animal Sciences, Zhejiang University. Four different colonies were used as four experimental replicates (referred as E1–E4). The laboratory worker bees were raised in cages (A cylinder with a top circle diameter of 5 cm, a bottom circle diameter of 7.5 cm, and a height of 10 cm) and incubated at 31°C ± 1°C and 75% ± 5% relative humidity (RH). Dead bees were counted and removed daily. All pollen and syrup supplied to workers were irradiated and sterilized.

### Preparation of Honey Bee Gut Bacteria for Artificial Colonization

Five nurse workers were sampled from a hive and placed in a sterile centrifuge tube. They were soaked in 75% ethanol for 3 min and then washed 3 times with sterile water. After that, their guts were immediately dissected with high-temperature sterilized tweezers under sterile conditions and homogenized together in 1 mL Phosphate Buffered Saline (PBS). The gut homogenate was centrifuged at 10,000 × *g* for 10 min and the supernatant were removed to eliminate the possible virus contamination. Then 1 mL PBS was used to resuspend the bacteria and 100 μL suspension was added and mixed with 2 mg sterilized pollen.

### Direct Treatment of Antibiotic on Honey Bee Workers

Antibiotic treated (AT) workers and conventional gut community (CV) workers were obtained using a protocol described by Raymann et al. (2017). Sealed brood combs containing emerging adult workers were removed from the same hive as above and placed in an incubator at 34°C ± 1°C with 80% ± 5% RH overnight. In the following day, newly emerged workers were randomly assigned to AT or CV groups (100 per cage). In each cage, workers were supplied with pollen containing honey bee gut microbiota for 5 days to establish a normal gut community. Then, AT workers were treated by providing *ad libitum* tetracycline solution at 450 μg/mL (450 μg/mL Tetracycline in filter-sterilized 0.5 M sucrose solution) for another 5 days, whereas CV workers were only fed with 0.5 M sucrose solution for 5 days. Afterward, bees were immobilized at 4°C and marked with red and green paint pens for the AT and CV workers, respectively, and then returned to their original colony. Two days later, 15 CV and 15 AT workers were sampled again, of which 5 were used for 16S rRNA sequencing, 5 for quantification of bacterial loads, and the remaining 5 workers were sampled to prepare normal or dysbiotic gut bacteria for downstream experiments.

### Evaluation of the Pass-on Effect of Honey Bee Gut Community Disturbance Induced by Antibiotic Treatment

Germ-free workers were obtained using the protocol described by Zheng et al. (2017). Briefly, late-stage pupae, with pigmented eyes but lacking movement, were removed from brood frames to sterile dishes using high-temperature sterilized tweezers. The dishes were placed in an incubator at 34°C ± 1°C with 80% ± 5% RH until bees emerged. These newly emerged germ-free bees were randomly assigned to 4 different treatment groups (30 workers per cage): (1) Workers colonized with normal gut community (referred as Nor); (2) Workers colonized with dysbiotic gut community (referred as Dys); (3) Workers colonized with normal gut community and treated with 10 μg/mL tetracycline (referred as NorA); (4) Workers colonized with dysbiosis gut community and treated with 10 μg/mL tetracycline (referred as DysA). On Day 6 post colonization, 5 workers from each group were sampled for 16S rRNA sequencing, 5 were used for quantification of bacterial loads, and rest of the workers were kept until Day 20 post colonization, to evaluate their survival rate.

### Gut Microbiota Analysis

Bacterial genomic DNA was extracted using the TIANamp Stool DNA Kit (Tiangen Biotech Co., Ltd., Beijing, China) according to the manufacturer’s protocol. Polymerase chain reactions (PCR) targeting the V4 regions of the 16S rDNA genes were carried out using the specific primer with the barcode. Sequencing libraries were generated using the NEBNext Ultra DNA Library Prep Kit (NEB, United States) following manufacturer’s recommendations and index codes were added. The library quality was assessed on the Qubit@ 2.0 Fluorometer (Thermo Fisher Scientific, Waltham, MA, United States) and Agilent Bioanalyzer 2100 system (Agilent Technologies, Santa Clara, CA, United States). The library was sequenced on an Illumina NovaSeq platform and 250 bp paired-end reads were generated. The 16S rRNA gene sequences were deposited in NCBI^1^ Sequence Read Archive with a BioProject accession number PRJNA225925.

Paired-end reads from the original DNA fragments were merged using FLASH software (Magoc and Salzberg, 2011). Sequences were analyzed using the Quantitative Insights into Microbial Ecology (QIIME) software package (Caporaso et al., 2010). We used pick_de_novo_otus.py to pick operational taxonomic units (OTUs) by making an OTU table. Sequences with ≥97% similarity were assigned to the same OTUs. We picked a representative sequence for each OTU and used the RDP classifier to annotate taxonomic information for each representative sequence. OTUs abundance information were normalized using a standard of sequence number corresponding to the sample with the least sequences. Subsequent analysis of alpha diversity and beta diversity were performed basing on the normalized data. Beta diversity was assessed using the Unweighted Pair-group Method followed by principal coordinate analysis (PCoA).

### Quantification of Bacterial Loads in the Gut of Honey Bees

Bacterial loads of workers were determined by quantitative PCR (qPCR) using universal bacterial 16S rRNA primers (F: 5′-AGAGTTTGATCCTGGCTCAG-3,′ R: 5′-CTGCTGCCTCCCGTAGGAGT-3′). Sampled guts of workers were obtained according to the protocol described by Wu et al. (2020a) and their DNA using TIANamp Stool DNA Kit (Tiangen Biotech Co., Ltd., Beijing, China) according to the manufacturer’s protocol. The StepOne Plus real time PCR system was used for the absolute quantification of 16S rRNA copy number. The thermal cycling conditions were as follows: the initial degeneration step was 40 amplification cycles of 95°C 30 s, 95°C 5 s and 60°C annealing 30 s. And melt curve analysis from 60°C to 95°C 0.5°C/5 s to determine the expected dissociation curve.

### Data Analysis

SPSS software version 22.0 (IBM SPSS Statistics; Chicago, IL, United States) and GraphPad Prism 8 (GraphPad Software, San Diego, CA, United States) were used for statistical analysis. After verifying variance homogeneity and data normality, independent sample *t-*test or Mann-Whitney test was used to calculate the statistical difference of bacterial load and the alpha diversity among groups. Survival analysis was performed using Kaplan-Meier survival curve and log-rank test. All graphs were generated in GraphPad Prism 8.

## Results

### Direct Exposure to Tetracycline Caused Significant Disruption of Gut Community of Honey Bee

**FIGURE 1:**
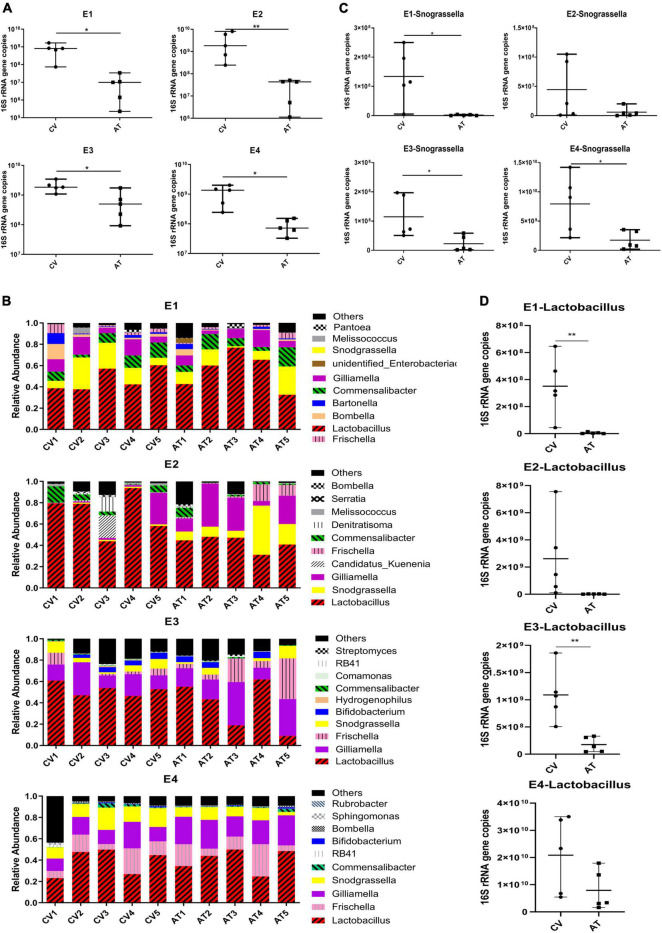
Changes of honey bee gut microbiota after tetracycline treatment. **(A)** The absolute abundance of gut bacteria of CV and AT workers (*n* = 5), total bacterial 16S rRNA gene copies were estimated by qPCR. **(B)** Stacked column graph showing the relative abundance of bacterial species in CV and AT workers. **(C)** The absolute abundance of *Snodgrassella* in CV and AT workers (*n* = 5). **(D)** The absolute abundance of *Lactobacillus* in CV and AT workers (*n* = 5). *p < 0.05, **p < 0.01, student’s *t*-test. CV, conventional gut community workers, AT, Antibiotic treated workers.

**FIGURE 2:**
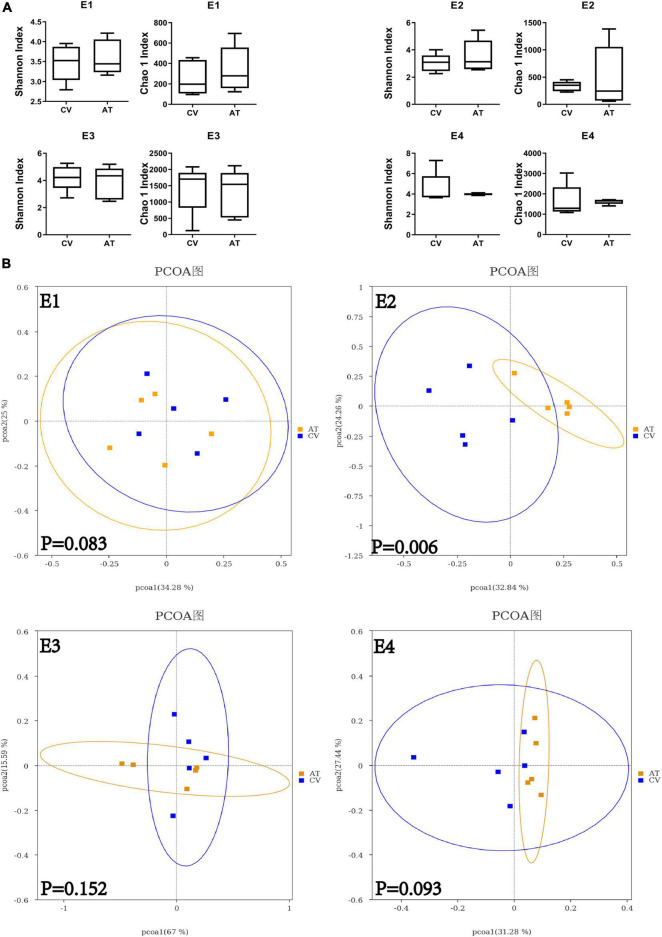
Alpha and beta diversity of CV and AT honey bee workers. **(A)** Difference in alpha diversity between CV and AT workers measured using Shannon index and Chao 1 index. **(B)** The principal coordinate analysis (bray-Curtis) in gut community between CV and AT workers. E1–E4 represent four experimental replicates with four different honey bee colonies.

The absolute abundance of gut bacteria was significantly reduced in antibiotic treated (AT) workers compared to conventional gut community (CV) workers in all the four replicates (Figure 1A, student’s *t*-test: for E1: *t* = 3.14 df = 8, *p* = 0.0138; for E2: *t* = 2.151, df = 8, *p* = 0.0079; for E3: *t* = 2.894, df = 8, *p* = 0.0201; for E4: *t* = 3.158, df = 8, *p* = 0.0134). 16s rDNA amplicon sequencing demonstrated that *Lactobacillus*, *Frischella*, *Commensalibacter*, *Bartonella*, *Snodgrassella* and *Gilliamella* are the core bacterial species dominating in CV workers and AT workers (Figure 1B). The application of tetracycline did not remove any of these core bacteria completely, but caused a general decreasing trend of these bacteria (Supplementary Figure 1). For example, the absolute abundance of *Snodgrassella* was significantly decreased in AT workers in E1, E3 and E4, and showed a decreasing trend in E2 (Figure 1C, student’s *t*-test: for E1: *t* = 3.159 df = 8, *p* = 0.0134; for E2: *t* = 1.689 df = 8, *p* = 0.1298; for E3: *t* = 2.669, df = 8, *p* = 0.0284; for E4: *t* = 2.656, df = 8, *p* = 0.029). The absolute abundance of *Lactobacillus* was significantly decreased in AT workers in E1 and E3, and showed a decreasing trend in E2 and E4 (Figure 1D, student’s *t*-test: for E1: *t* = 3.464, df = 8, *p* = 0.0085; for E2: *t* = 1.920, df = 8, *p* = 0.0912; for E3: *t* = 3.977 df = 8, *p* = 0.0041; for E4: *t* = 1.809 df = 8, *p* = 0.1080). However, no significant difference was found on the alpha diversity (Shannon index and Chao 1 index) of microbiota species between CV workers and AT workers in any of the replicates (Figure 2A). Principal coordinate analysis (PCoA, using bray-curtis distance) showed that gut community compositions of AT workers were dramatically changed in contrast to CV workers in E2 (Figure 2B, Anosim analysis, *p* = 0.006), but not in other 3 replicates.

### Tetracycline-Induced Gut Microbiome Disequilibrium Passed to Their Receivers

**FIGURE 3:**
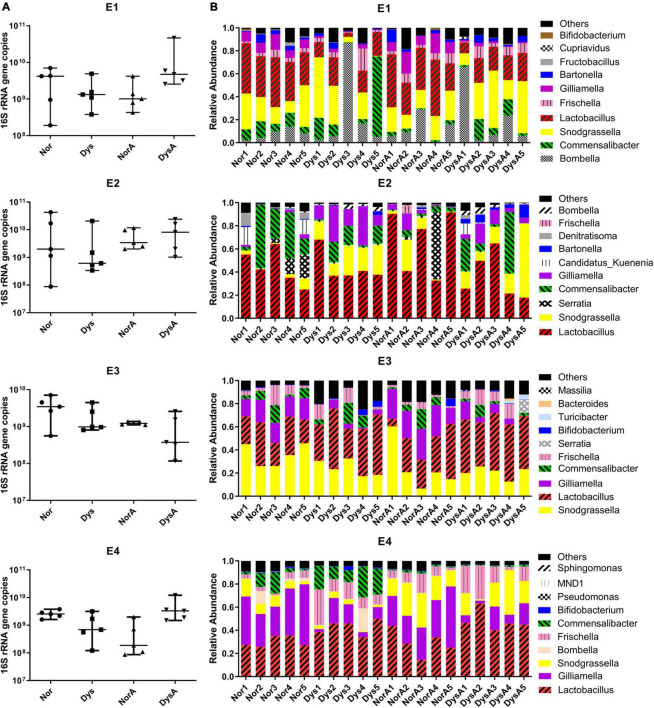
Size and Composition of gut bacteria of Nor, Dys, NorA, and DysA honey bee workers. **(A)** The absolute abundance of gut bacteria of Nor, Dys, NorA and DysA workers (*n* = 5), total bacterial 16S rRNA gene copies were estimated by qPCR. **(B)** Stacked column graph showing the relative abundance of bacterial species in Nor, Dys, NorA, and DysA workers. Nor, workers colonized with normal gut community, Dys, workers colonized with dysbiotic gut community, NorA, workers colonized with normal gut community and treated with 10 μg/mL tetracycline, DysA, workers colonized with dysbiotic gut community and treated with 10 μg/mL tetracycline.

**FIGURE 4:**
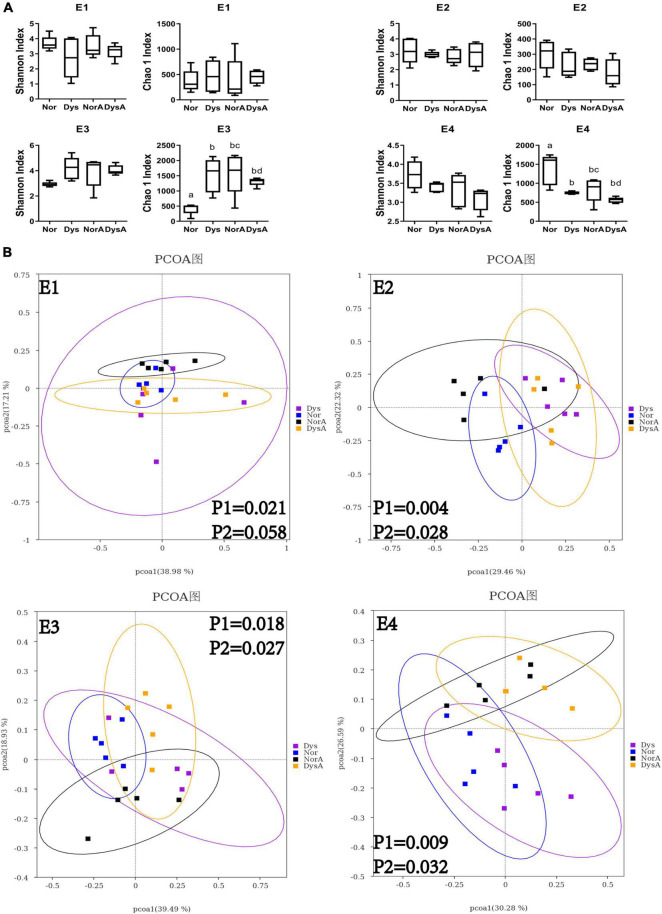
Alpha and beta diversity of Nor, Dys, NorA and DysA honey bee workers. **(A)** Difference in alpha diversity between Nor, Dys, NorA and DysA workers measured using Shannon index and Chao 1 index. **(B)** The principal coordinate analysis (bray-Curtis) in gut community between Nor, Dys, NorA, and DysA workers. E1–E4 represent four experimental replicates with four different colonies.

To reveal the possibility of pass-on effect to their nestmates of tetracycline-treated gut microbiota disequilibrium, we determined the gut microbiota composition and size of workers that were exposed to either normal or dysbiotic gut community. Quantification with 16s rDNA copy numbers revealed that the total bacterial abundances of these workers were not influenced by the type of gut microbiota they received (Figure 3A), but 16s rDNA amplicon sequencing analyses of the gut microbiota revealed that the compositions of their gut community were significantly influenced. The overall community structure showed that the gut microbiome of these workers in different treatment groups were dominated by six genera in our samples, namely, *Lactobacillus*, *Bifidobacterium*, *Snodgrassella*, *Bartonella*, *Gilliamella*, and *Frischella* (Figure 3B). The species diversity comparisons with Shannon index did not show significant difference between the groups of Workers colonized with normal gut community (Nor) and Workers colonized with dysbiotic gut community (Dys) (Figure 4A) in any of the four replicates, while species richness comparisons with Chao 1 index showed significant difference between the two groups in two replicates (E3 and E4) (student’s *t*-test: for E3: *t* = 3.464, df = 8, *p* = 0.0085; for E2: *t* = 1.920, df = 8, *p* = 0.0912). There were no significant differences in Shannon index and Chao 1 index between the NorA and the DysA in the four experiments. PCoA analysis revealed that the overall microbiota structure was significantly different according to the type of gut bacteria they received, as samples of Nor and Dys formed two distinctive cluster in three of the four replicates (Figure 4B, Anosim analysis: for E1: *p*1 = 0.021, for E2: *p*1 = 0.004, for E3: *p*1 = 0.118, for E4: *p*1 = 0.009) and samples of NorA and DysA formed two distinctive cluster in two of the four replicates (Figure 4B, Anosim analysis: for E1: *p* = 0.058, for E2: *p* = 0.028, for E3: *p* = 0.027, for E4: *p* = 0.302).

To evaluate the possible negative impact of low dosage antibiotic residue on the establishment of honey bee gut microbiota, 10 μg/mL tetracycline was used to treat workers during the development of their gut communities. However, no significant impact was observed, as neither the community size nor the makeup was significantly different between Nor and NorA, or between Dys and DysA (Figures 3A, 4A).

In addition, we evaluated and compared the longevity of workers from different group, and the survival rates of bees of Dys, NorA or DysA were not significantly different from that of control group (Supplementary Figure 2).

**FIGURE 5:**
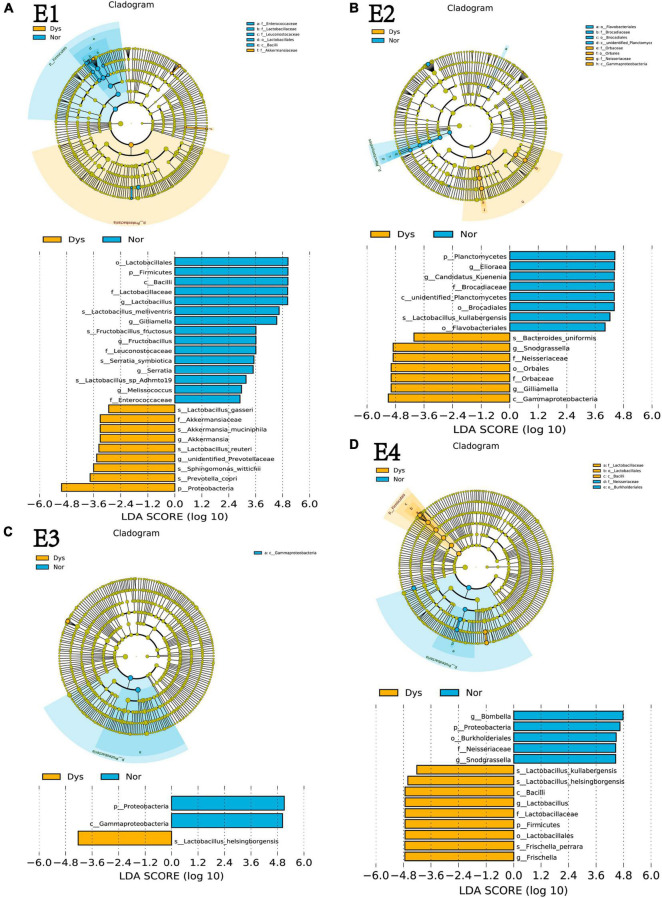
The main taxa that were different in Nor vs Dys in E1 **(A)**, E2 **(B)**, E3 **(C)** and E3 **(D)** on the basis of LEFSe analysis (taxa with >4). Blue represents taxa that significantly higher in Nor bees and yellow represents taxa that significantly higher in Dys bees. E1–E4 represent four experimental replicates with different colony. Nor: workers colonized with normal gut community, Dys, workers colonized with dysbiotic gut community.

The linear discriminant analysis effect size (LEfSe) algorithm was then applied to determine which microbiota taxa caused the significant difference between Nor workers and Dys workers. Significant differences on the relative abundances of some taxa were found, but the taxa showing differences were not consistent in the four replicates (Figures 5A–D). For example, in E1, Dys workers showed higher phylum_Proteobacteria relative abundance, but lower phylum_Firmicutes and genus_*Gilliamella* relative abundance. In E2, Dys workers harbored lower genus_*Snodgrassella* and genus_*Gilliamella* relative abundance. In E3, Dys workers harbored higher class_Gammaproteobacteria and phylum_Proteobacteria relative abundance. And in E4, Dys workers harbored higher phylum_Firmicutes, genus_*Lactobacillus* and genus_*Frischella* relative abundance but lower phylum_Proteobacteria, genus_*Snodgrassella* and genus_*Bombella*.

## Discussion

Antibiotics have been widely used in animals for disease control and have significantly contributed to the development of animal husbandry industries. However, the collateral damage caused by antibiotic treatment has also been a great concern (Holzheimer, 2001; Martel et al., 2006; Modi et al., 2014; Becattini et al., 2016). The application of antibiotics in honey bees may not only cause damage to the individuals that received the application but also to their nestmates *via* certain pathways due to the sociality of honey bees. Therefore, we investigated the effect of honeybee gut microbiota dysbiosis caused by tetracycline treatment on the establishment of gut community of their nestmates.

Tetracycline treatment had persistent effects on both the size and composition of *A. mellifera* gut microbiota and reduced genetic diversity of core species in *A. mellifera* gut community (Raymann et al., 2017, 2018). In our study, tetracycline treatment also significantly reduced the absolute abundance of gut bacteria in honey bee workers. At the same time, tetracycline treatment significantly changed the overall microbiota structure in E2, and the same trend was observed in the other 3 replicates. These results suggest that both the CV and AT worker models were successfully established for the downstream experiments. However, unlike previous findings (Li et al., 2017; Raymann et al., 2017), the application of tetracycline did not cause significant difference in microbiota diversity within individual hosts (alpha diversity) in our study. This may be explained by the difference of incidence of antibiotic resistance, as the usage of antibiotics could be different between regions and apiaries.

The major contributors of honey bee microbiota are transmitted *via* direct contact during the first social interactions with hive mates (Engel and Moran, 2013; Powell et al., 2014). The gut microbiota of tetracycline-treated honey bee workers did not recover after being reintroduced to their colonies (Raymann et al., 2017), indicating a high possibility of cascade effect to their nestmates of the tetracycline treatment. We investigated this cascade effect in lab condition *via* artificially inoculating tetracycline-treated gut microbiota to newly emerged workers. Despite non-significant effect on the absolute amount and alpha diversity of receiver’s gut microbiota, beta diversity was found to be significantly altered in Dys workers compared to the Nor workers inoculated with normal gut microbiota in 3 of the 4 replicates. This result suggests that the impact of tetracycline treatment on gut microbiota may be transmitted to other individuals. Various functions have been found for each of the core species of honey bee gut bacteria (Raymann et al., 2017) and functional redundancy among them stabilize the general function of gut microbiota in honey bees (Wu et al., 2021). Therefore, the disruption of gut microbiota indirectly caused by tetracycline treatment may negatively affect the function of gut microbiota and subsequently the physiology of their host, despite that no difference was observed on the mortality of different groups in our study.

LEfSe analysis identified variable microbial taxa that differentiated the groups in each replicate, suggesting variations of this cascade effect among cases. The presence of the core species is quite consistent in honey bee gut microbiota, while their relative abundances vary at individual and colony levels in honeybees (Powell et al., 2014; Dong et al., 2020). It is therefore not surprising that the results vary among replicates. This also suggests that multiple sources of gut microbiota and honey bees are necessary in this kind of research on honey bee gut microbiota.

The antibiotic residue in bee hive is inevitable after application. A previous study has shown that the tetracycline hydrochloride could be detected in honey 146 days after treatment in a colony (Martel et al., 2006). Thus, the possible negative impact of low concentration antibiotic on the development of honey bee gut microbiota and survival rate of bees is worthy of attention. No significant effect was found in this study possibly due to the low dosage treatment and/or the antibiotic resistance of bacteria. Interestingly, when the low dosage treatment was applied simultaneously, beta diversity was also found to be significantly altered in 2 of the 4 replicates when bees received tetracycline-treated gut microbiota. This further confirmed the pass-on effect of high dosage tetracycline treatment.

## Conclusion

Our findings clearly demonstrated that direct exposure to tetracycline caused significant disruption of gut community of honey bee workers and the disorder has a cascade effect on the workers inoculated with the antibiotic-treated gut microbiota. This pass-on effect of antibiotic treatment represents a new negative impact of antibiotics in honey bee colonies and further study is needed to monitor the constant exposure to antibiotic along with its potential damage to honey bee health.

## Data Availability Statement

The datasets presented in this study can be found in online repositories. The names of the repository/repositories and accession number(s) can be found in the article/Supplementary Material.

## Author Contributions

SJ, YW, FH, and HZ conceived and designed the study. SJ, YW, GC, and SW performed experiments. SJ and YW analyzed data and drafted the manuscript. All authors edited and approved the final version of the manuscript.

## Conflict of Interest

The authors declare that the research was conducted in the absence of any commercial or financial relationships that could be construed as a potential conflict of interest.

## Publisher’s Note

All claims expressed in this article are solely those of the authors and do not necessarily represent those of their affiliated organizations, or those of the publisher, the editors and the reviewers. Any product that may be evaluated in this article, or claim that may be made by its manufacturer, is not guaranteed or endorsed by the publisher.

## Footnotes

1
https://www.ncbi.nlm.nih.gov/
