# Supplementary material for: The Pass-on Effect of Tetracycline-Induced Honey Bee (Apis mellifera) Gut Community Dysbiosis
Source: Front Microbiol. 2022 Jan 18;12:781746. doi: 10.3389/fmicb.2021.781746 (PMC8804527; doi:10.3389/fmicb.2021.781746)
Supplement: Supplementary file 1 [file Data_Sheet_1.docx]

**Supporting information**


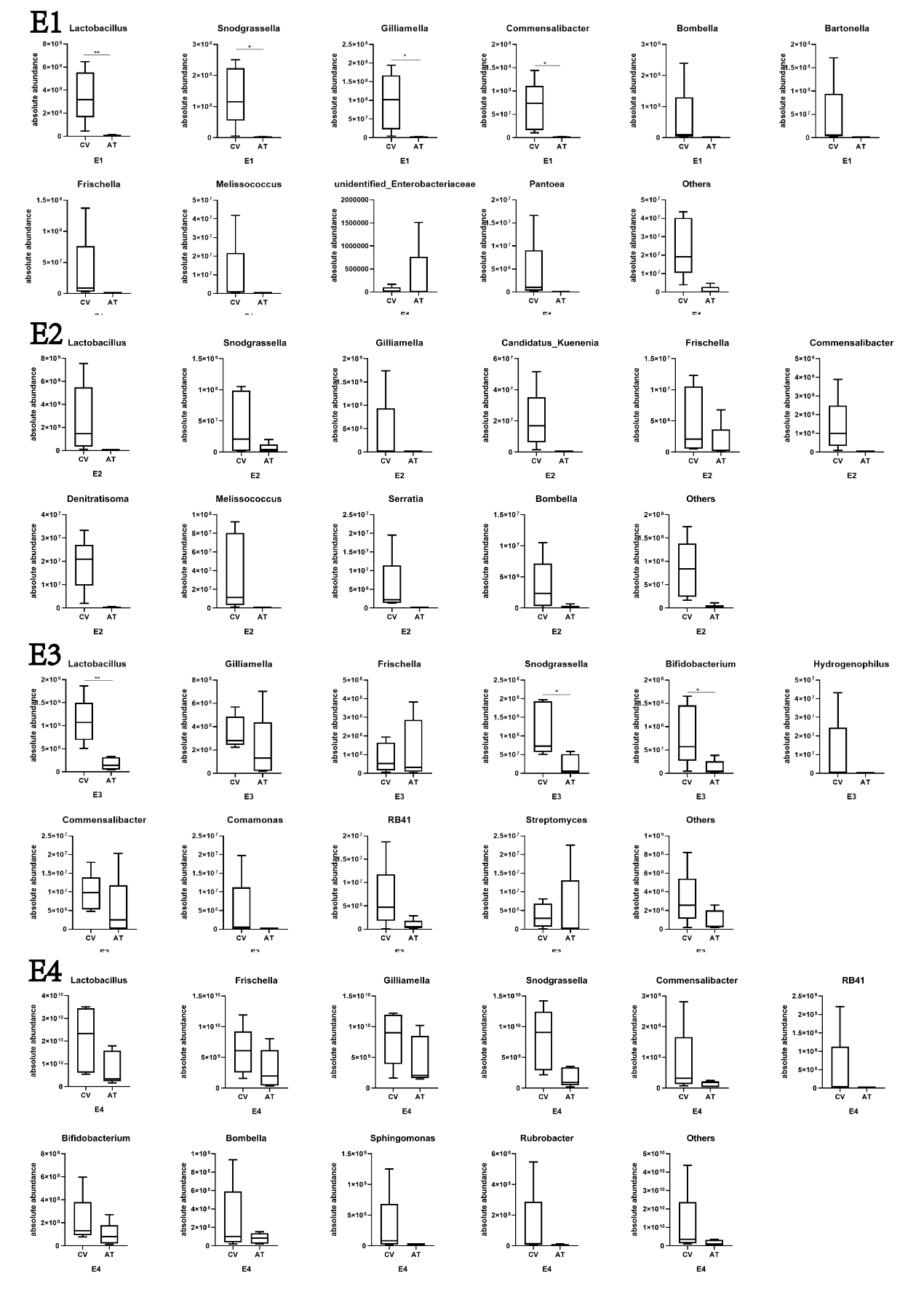


Supplement Fig.1: Boxplot of the absolute abundance of gut bacteria of CV and AT workers at the genus level. The 10 most abundant genera and others for bacteria are shown. CV: conventional gut community workers, AT: Antibiotic treated workers.


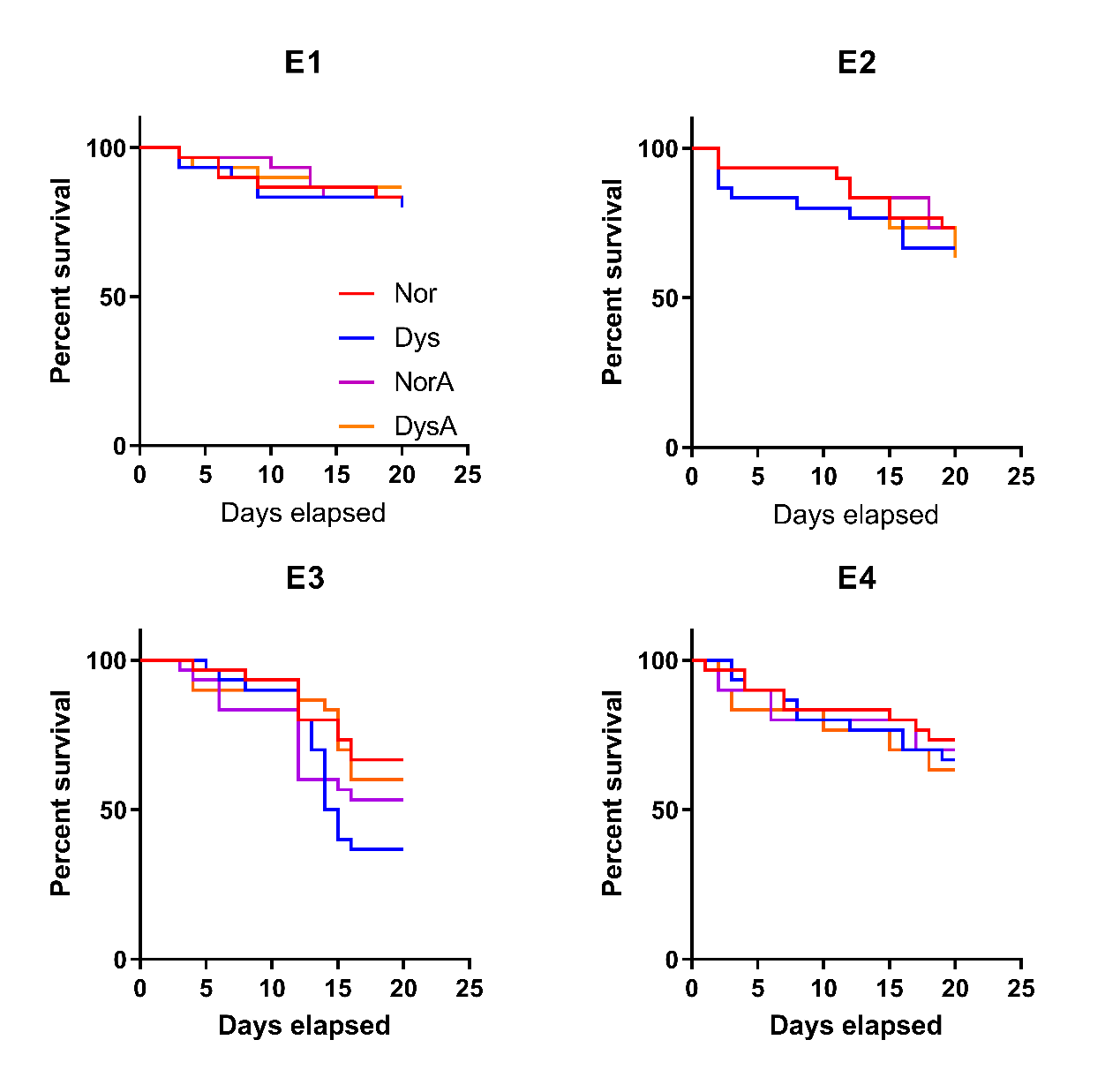


Supplement Fig.2: The longevity of workers under different treatment. E1-E4 represent four experimental replicates with four different colonies. Nor: workers colonized with normal gut community, Dys: workers colonized with dysbiotic gut community, NorA: workers colonized with normal gut community and treated with 10 μg/mL tetracycline, DysA: workers colonized with dysbiotic gut community and treated with 10 μg/mL tetracycline. E1-E4 represent four experimental replicates with four different colonies.
